# Therapeutic Implications of Dietary Polyphenols-Loaded Nanoemulsions in Cancer Therapy

**DOI:** 10.1021/acsabm.3c01205

**Published:** 2024-03-25

**Authors:** Ritu Tomar, Sabya Sachi Das, Venkata Krishna Rao Balaga, Srusti Tambe, Jagannath Sahoo, Santosh Kumar Rath, Janne Ruokolainen, Kavindra Kumar Kesari

**Affiliations:** † School of Pharmaceutical and Population Health Informatics, DIT University, Dehradun, Uttarakhand 248009, India; ‡ School of Pharmacy, Suresh Gyan Vihar University, Mahal Road, Jagatpura, Jaipur, Rajasthan 302017, India; § Department of Pharmaceutical Science & Technology, Institute of Chemical Technology, Mumbai, Maharashtra 400019, India; ∥ Shobhaben Pratapbhai Patel School of Pharmacy & Technology Management, SVKM’S NMIMS, V. L. Mehta Road, Vile Parle (W), Mumbai, Maharashtra 400056, India; ⊥ Department of Applied Physics, School of Science, Aalto University, Espoo 00076, Finland

**Keywords:** Cancer, Cancer therapy, Biodegradable, Biocompatible, Nanoemulsions, Polyphenols, Targeted delivery

## Abstract

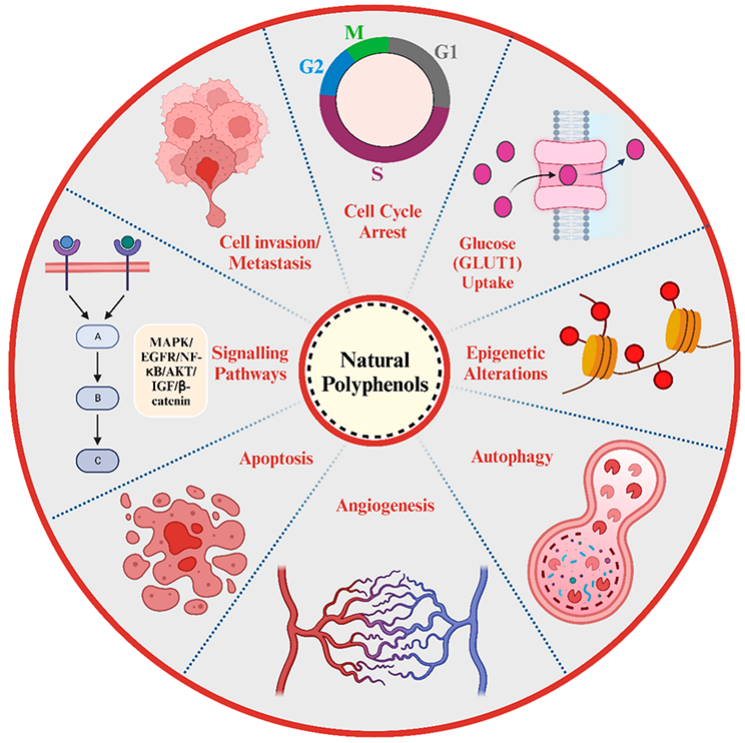

Cancer is one of the major causes of death worldwide, even the second foremost cause related to non-communicable diseases. Cancer cells typically possess several cellular and biological processes including, persistence, propagation, differentiation, cellular death, and expression of cellular-type specific functions. The molecular picture of carcinogenesis and progression is unwinding, and it appears to be a tangled combination of processes occurring within and between cancer cells and their surrounding tissue matrix. Polyphenols are plant secondary metabolites abundant in fruits, vegetables, cereals, and other natural plant sources. Natural polyphenols have implicated potential anticancer activity by various mechanisms involved in their antitumor action, including modulation of signaling pathways majorly related to cellular proliferation, differentiation, relocation, angiogenesis, metastatic processes, and cell death. The applications of polyphenols have been limited due to the hydrophobic nature and lower oral bioavailability that could be possibly overcome through encapsulating them into nanocarrier-mediated delivery systems, leading to improved anticancer activity. Nanoemulsions (NEs) possess diverse feasible properties, including greater surface area, modifiable surficial charge, higher half-life, site-specific targeting, and formulation imaging capability necessary to create a practical therapeutic impact, and have drawn increased attention in cancer therapy research. This review has summarized and discussed the basic concepts, classification, delivery approaches, and anticancer mechanism of various polyphenols and polyphenols-encapsulated nanoemulsions with improved cancer therapy.

## 1 Introduction

The complexity of cancer’s diversity, which includes genetics, cell and tissue biology, pathology, and therapeutic response, is intimidating.^1^ Cancer is marked by uncontrolled cell growth and the development of metastatic properties.^2^ A wide-range of literature reported about numerous symptoms of cancer-related disorders is being generated by ever more powerful experimental and computational instruments and technologies.^3^ Cancer is also classified as a developmental abnormality, as it disrupts the normal development of cells regarding differentiation and proliferation.^4−8^ Cancer cells typically possess several cellular and biological processes including, persistence, propagation, differentiation, cellular death, and expression of cellular-type specific functions. Unfortunately, the control of key components of cellular function has been disrupted.^9^ In most of the situations, activation of oncogene(s) and/or deactivation of tumor suppressor genes leads to unrestrained cellular cycle progression and deactivation of the apoptosis process.^10,11^ Compared with benign tumors, malignant cancers develop metastasis, partly facilitated by downregulation of cell adhesion receptors entailed for tissue-specific cellular-cellular association and receptors upregulation that specifically boosts cellular motility.^2^

Cancer development and progression is a complex process involving various epigenetic alterations, including variations in the histone acetylation and DNA methylation, genomic mutational development leading to rehabilitated gene expressions, and also affecting overall cellular functionality within the normal cell.^12^ These phenotypic changes make the normal transitional cell cancerous, eventually producing a malignant phenotype.^9^ The major cause for the necessity of developing numerous new diagnostic and therapeutic platforms is to unravel and understand various molecular mechanisms associated with malignant alterations and metastatic progressions in carcinogenic cells.^9^ According to research, substantial genetic modifications may emerge early in the natural history of a tumor.^13^ A greater understanding and exploration of pharmacological conditions associated with carcinogenesis might allow one to establish more sensitive testing tools and targeted therapeutic anticancer strategies, based on virulence mechanisms.^13^ The molecular picture of carcinogenesis and progression is unwinding, courtesy of modern technologies, and it appears to be a tangled combination of processes occurring within and between cancer cells and their surrounding tissue matrix.^9^

Tumors can originate and progress due to the loss of tumor suppressor activity, the stimulation of oncogene functions, or both.^14^ The major cellular mechanisms involved with the alterations in tumor suppressor genes and oncogene vary among tumor histology’s and may even differ between patients with the same histology.^14^ For example, chromosomal rearrangements that activate multiple oncogenes are involved in developing several soft-tissue sarcomas and papillary thyroid carcinomas. In contrary, the beginning of several colon and pulmonary cancer types are demonstrated to entail oncogenes and tumor-specific suppressor changes.^14^ Cancerous cell growth and progression start with the tumor initiation steps, which are followed by various steps in the tumor cell proliferation stage.^15^ Tumor initiation begins with the first cell to show growth dysregulation. The process is assumed to necessitate at least two genomic changes, leading to the loss of cellular capability to alleviate the operational deficiency and becomes immortal as a result.^16^ If the progeny cells survive, they may evolve into a progressive clonal populace, leading to the formation of the primary tumor, and eventually leading to the main tumor.^16^

The lack of normal cell proliferation restraint is the first and most visible symptom observed during the progression of cancer.^17^ Inhibition of regular contact occurs when cells multiply until they attain a finite mass, which is identified and established by the presence of specific growth factors; however, the cancer cells do not exhibit this behavior.^17^ Suggested as a factor in metastatic cell growth and survival in ectopic sites is if the cells fail to experience apoptosis under a condition of scarcity.^18^ Cancer cells ignore the cellular signaling that tells normal and healthy cells to stop proliferating and move into the cell cycle’s G_0_ phase, continuing to expand above the typical density ratio.^18^ Senescence and apoptosis are ordinarily tightly regulated processes that are severely disturbed. The progression of these anomalies leads to the clinically significant malignant phenotype.^3,18^ As the tumor grows, more mutations occur, resulting in a diverse cell population. New phenotypes that predict lower apoptosis rates, quicker division rates, low metabolic necessities, improved capacity to attract neo-vasculatures, and metastatic competencies acquire an advantage and eventually account for a larger extent of the tumor size.^19,20^

Polyphenols are plant secondary metabolites that are abundant in fruits, vegetables, cereals, and drinks. Because of their possible beneficial impacts on human health, polyphenols and other dietary phenolics are attracting growing scientific attention.^21^ Epidemiological studies and meta-analyses strongly suggest that long-term consumption of a plant polyphenol-rich diet may protect against several ill health conditions, including malignancy, cardiovascular and neurological complications, diabetes, and osteoporosis.^22,23^ In various plant species, over 8000 polyphenolic chemicals have been found and nearly 4000 flavonoids have been discovered among them.^24^

The applications of polyphenols have been limited due to their hydrophobic nature and lower oral bioavailability, which can be overcome by encapsulating them into nanoformulations leading to improved anticancer activity.^25,26^ The creation of nanosystems to improve the physicochemical stability of flavonoids can be done using novel techniques made possible by nanotechnology.^27,28^ Both oil-in-water (O/W) and water-in-oil (W/O) nanoemulsions (NEs), but particularly O/W types, have been shown to be very successful for the delivery of various lipophilic drugs, enhancing the *in vitro* activity of chemotherapeutic agents, or improving the bioavailability of flavonoids, but can only encapsulate drugs with similar lipophilicity.^29,30^ Water-in-oil-in-water (W/O/W) NEs strategies containing lipophilic, hydrophilic, and amphiphilic molecules were developed to get around this restriction.^31^ Growing interest recently has been seen in using the principles of structural design to increase the functional performance of products based on emulsions.^32^ Traditional microencapsulation techniques cease to provide polyphenols stability in physiological environments, because of their greater particle size, lower zeta potential, and also lower drug entrapment efficiency.^32^ Thus, herein in this review work we have summarized and discussed the basic concepts, classification, delivery approaches, and anticancer mechanism of various polyphenols and polyphenols-encapsulated NEs with improved cancer therapy.

## 2 Nanoemulsion: a Potential Carrier for Delivering Polyphenols in Cancer therapy

**Figure 1:**
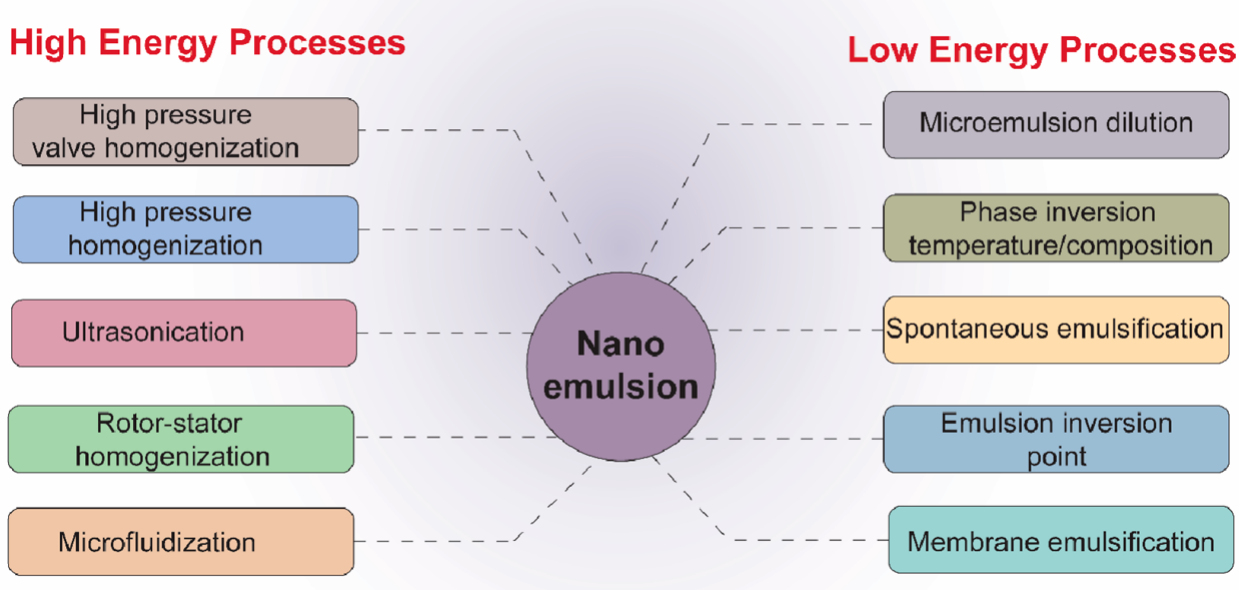
Various high and low energy processes for manufacturing nanoemulsion-based drug delivery strategies.

Nanoemulsions are a class of drug delivery system, mostly used to deliver compounds with low water solubility.^32,33^ NEs are dispersions of either O/W or W/O in colloidal forms, where an appropriate surfactant stabilizes the two immiscible liquids.^34^ NEs are further classified as multiphase NEs (W/O/W) and biphasic (O/W or W/O) based upon the composition and relative distribution of the continuous dispersion medium and the internal dispersed phase. The proportional volumes of internal and external mediums in a NE are measured using the phase volume ratio (Φ), which also determines its droplet number and overall stability.^35^ The NEs can be designed and fabricated using various high and low energy processes (Figure 1).^36,37^

Nanoemulsion is mainly a type of a colloidal dispersion, that usually contains an oil(s) phase, surfactant(s), cosurfactant(s), and an aqueous phase, while its core determines the influence over the drug’s therapeutic effect, globular size, and other physico–chemical properties, along with its stability.^34,38^ Unlike coarse emulsions which have a milky white color appearance (due to the presence of micron size droplets that participate in multiple light scattering), colloidal emulsions exhibit a clear or cloudy appearance because of the small droplet size (mean droplet diameter less than 500 nm).^39,40^ Moreover, the encapsulation further increases the plasma half-life and protects the drug from degradation.^41^ The optimal concentration of emulsifiers or surfactants should lower the interfacial tension, absorb quickly at the interface, and use stearic or electrostatic interactions to stabilize the surface.^30^ An amphiphilic molecule, such as phospholipids, polysaccharides, amphiphilic proteins, surfactants, or polymers are a few examples of emulsifiers. PEG-modified NEs allow prolonged circulation times and selective targeting.^30^ Additional options include ripening retarders, weighing agents, and texture modifiers. Nonionic surfactants such as Spans (sorbitan fatty acid esters) could also be employed.^30^

Either a spontaneous emulsification process, like that of a self-nanoemulsifying drug delivery system (SNEDDS), or a high-energy dispersion approach can be used to prepare NEs.^42^ When poorly water-soluble drugs are prepared as NEs, they possess superior both *in vivo* bioavailability and *in vitro* dissolution.^42^ As a result, for poorly soluble anticancer drugs, NEs have emerged as a viable drug delivery method.^43,44^ NEs can be formulated into variety of biphasic delivery systems including creams, gels, sprays, foams, aerosols, foams and can equally be delivered employing various routes such as oral, topical, transdermal, nasal, intravenous, ocular, and pulmonary.^35^ The properties of NEs such as greater surface area, modifiable surface charge, higher circulation half-life, precise targeting, and formulation imaging capability that are necessary to create an effective therapeutic impact have drawn increased attention in the field of cancer therapy research.^45^

**Figure 2:**
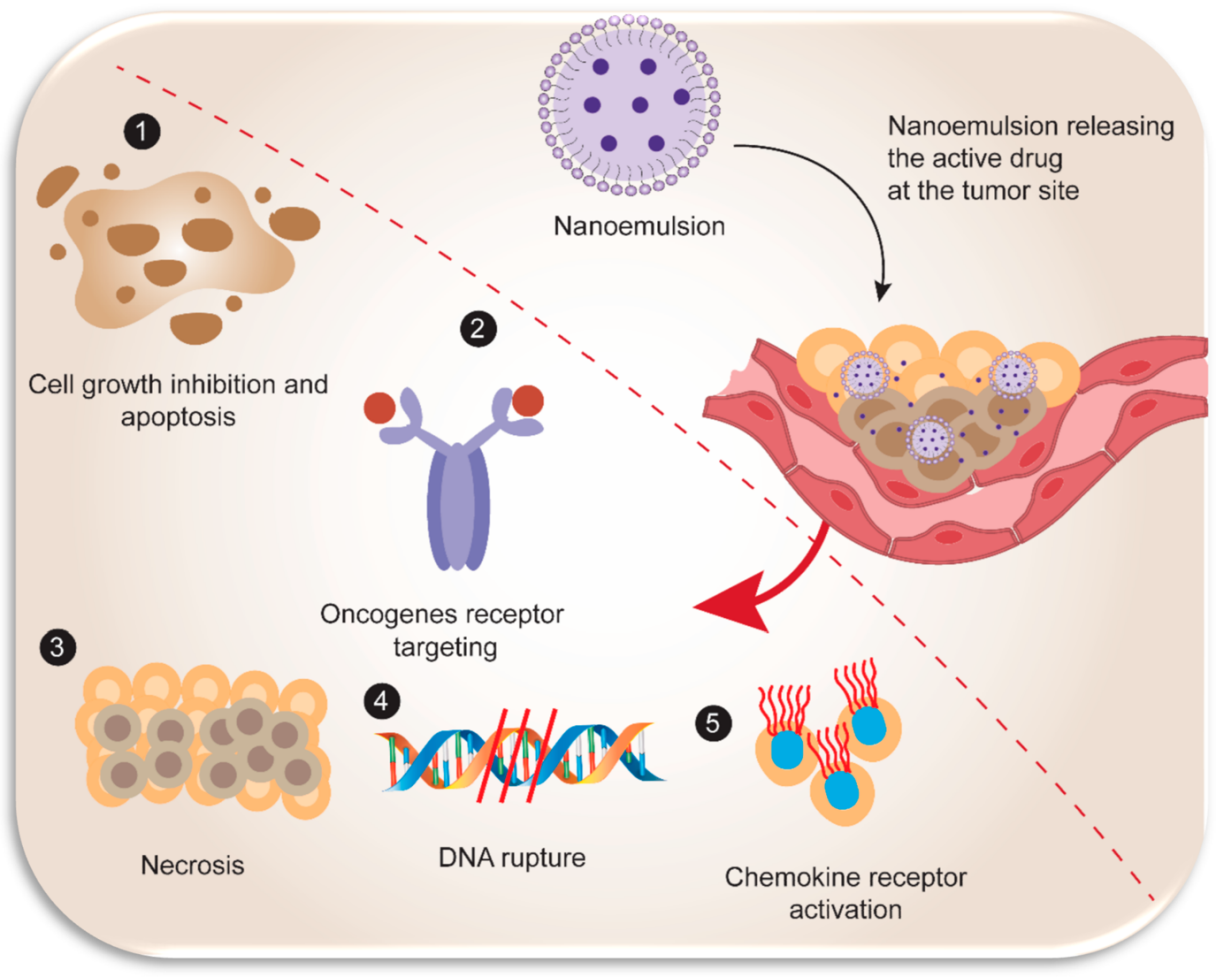
Schematic representation showing the general mechanism of targeted delivery of drug encapsulated in nanoemulsion. The major anticancer mechanism includes (1) cell growth inhibition and apoptosis; (2) oncogene receptor targeting; (3) necrosis; (4) DNA targeting and rupturing nucleic acid sequence; and (5) chemokine and cytokine receptor activation triggering pro-/anti-inflammatory cellular and molecular responses.

NEs, because of their versatile characteristic features, have been a preferred choice of researchers in treating several types of cancer including colorectal,^46^ breast,^47^ ovarian,^48^ lung,^49^ brain,^50^ leukemia,^51^ prostate,^52^ and melanoma.^53^ NEs ranging in size from 20 to 100 nm can get encapsulated and accumulate in tumor tissues (Figure 2) because they are both large enough to evade rapid renal clearance and small enough to flow through blood arteries.^54^ The probability of opsonisation by the mononuclear phagocytic systems (MPS) does, however, rise with this range of sizes.^54^ NEs can readily concentrate in vascularized tissues that surround cancer cells because of their particle size and capability to permeate through barriers and can be modified based on the type of drug(s) encapsulated, and site-specific targets.^45^ The technique by which ligands attached to the surface of NEs can identify a specific molecule on the tumor tissue is referred to as active targeting.^55^ Additionally, it exploits the surroundings of the tumor. Because it also creates a novel approach of delivering the medicine precisely to specific carcinogenic cells, it is more effective than passive targeting.^55^ This issue can be avoided by coating the NEs with hydrophilic polymers.^56^ Phosphatidyl-serine, a negatively charged molecule found on the surface of tumor cells, makes positively charged particulates the most prospective to be preserved by cancer cells for extended periods.^57^ Nevertheless, passive targeting cannot distinguish malignant tissues from healthy tissues.^58^ Comparing NEs to other drug delivery systems, their primary advantage is that they could be engineered to selectively target the tumor or cancerous cells while preventing multidrug resistance (MDR) situations.^30^ Delivery by passive targeting relies on the ERP effect, which is prevalent in tumor tissues. Since active targeting employs targeting moieties for cancer cells in addition to the EPR effect, it may add even more beneficial characteristics to the formulation.^59^ Compounds that resist MDR mechanisms can coencapsulate, or bind, to the surface of multifunctional NEs.^15,17,30^

## 3 Classification of Various Polyphenols and Their Role in Cancer Therapy

Polyphenol’s diversity and vast dispersion in plants have resulted in various classifications for these naturally occurring chemicals. Polyphenols have been categorized based on their chemical structure, biological function, and source of origin. Additionally, the bulk of plant-mediated polyphenols are primarily found in the form of glycosides with several sugar units and acylated sugars that are arranged at different positions throughout the polyphenol skeleton.^25,26^

**Figure 3:**
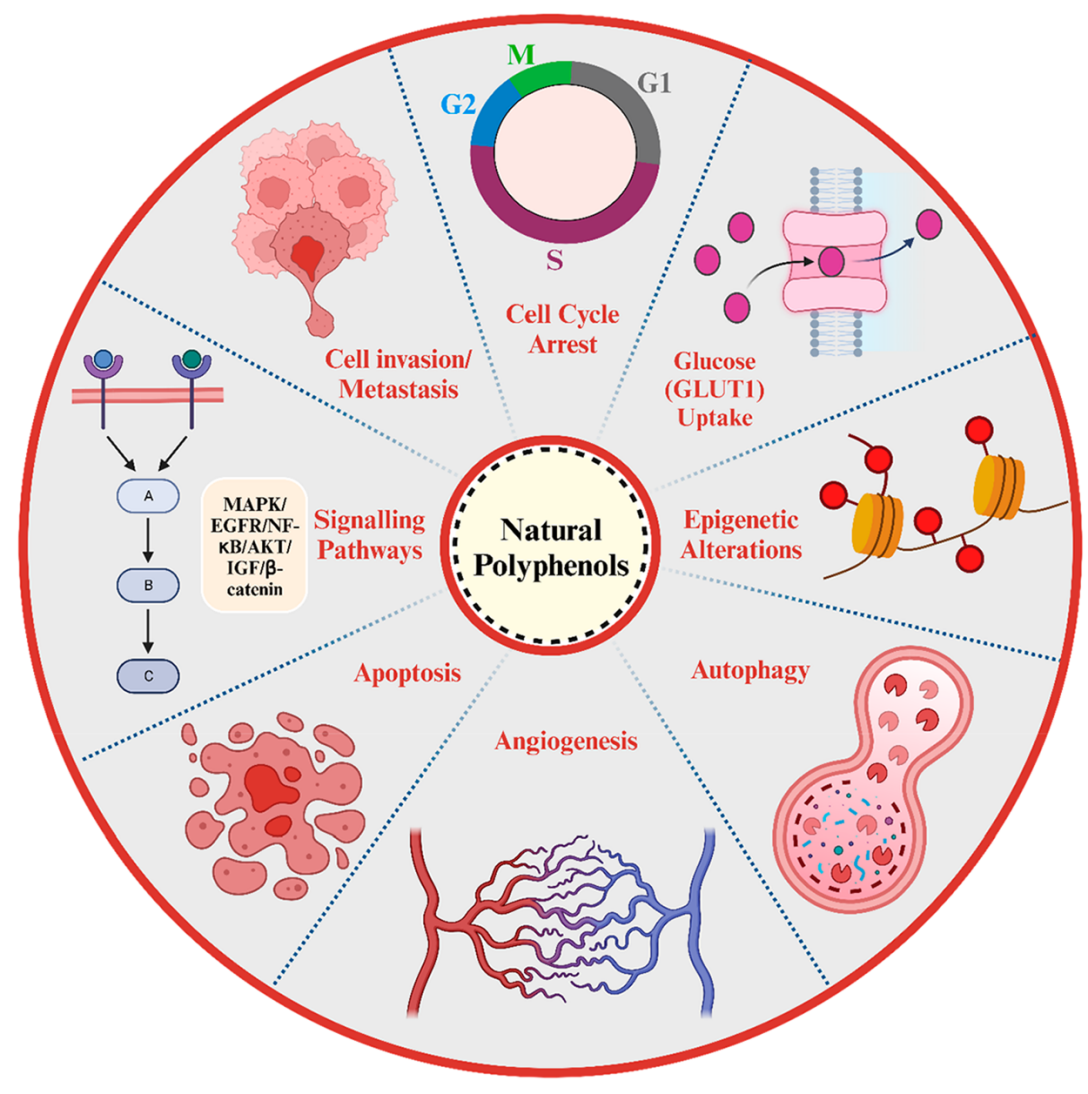
Schematic illustration representing different biological mechanisms involved in the chemotherapeutic potentials of natural dietary polyphenols. Created with BioRender.com.

Chemically, the polyphenols are defined as phytocompounds having phenolic structure-based properties and exhibit diverse multiple subtypes, including flavonoids, phenolic acids, lignans (LIG), and stilbenes (STB), that are also further individually classified in various subgroups.^25,26,60^ In accordance to epidemiologic evidence, a food intake having a high content of fruits and vegetables could minimize the probability of occurrence of specific cancers.^61^ Natural polyphenols have implicated potential anticancer or antitumor activity due to various biological mechanisms associated with different signaling pathways such as cell propagation, differentiation, relocation, angiogenesis, metastasis, and cellular death (Figure 3). These phytocompounds have shown the capability to specifically inhibit the growth or kill carcinogenic cells due to dysregulation of death processes that are typically associated with cancer etiopathology and thus can be used as potential chemotherapeutic agents.^60,62^

### 3.1 Flavonoids

**Figure 4:**
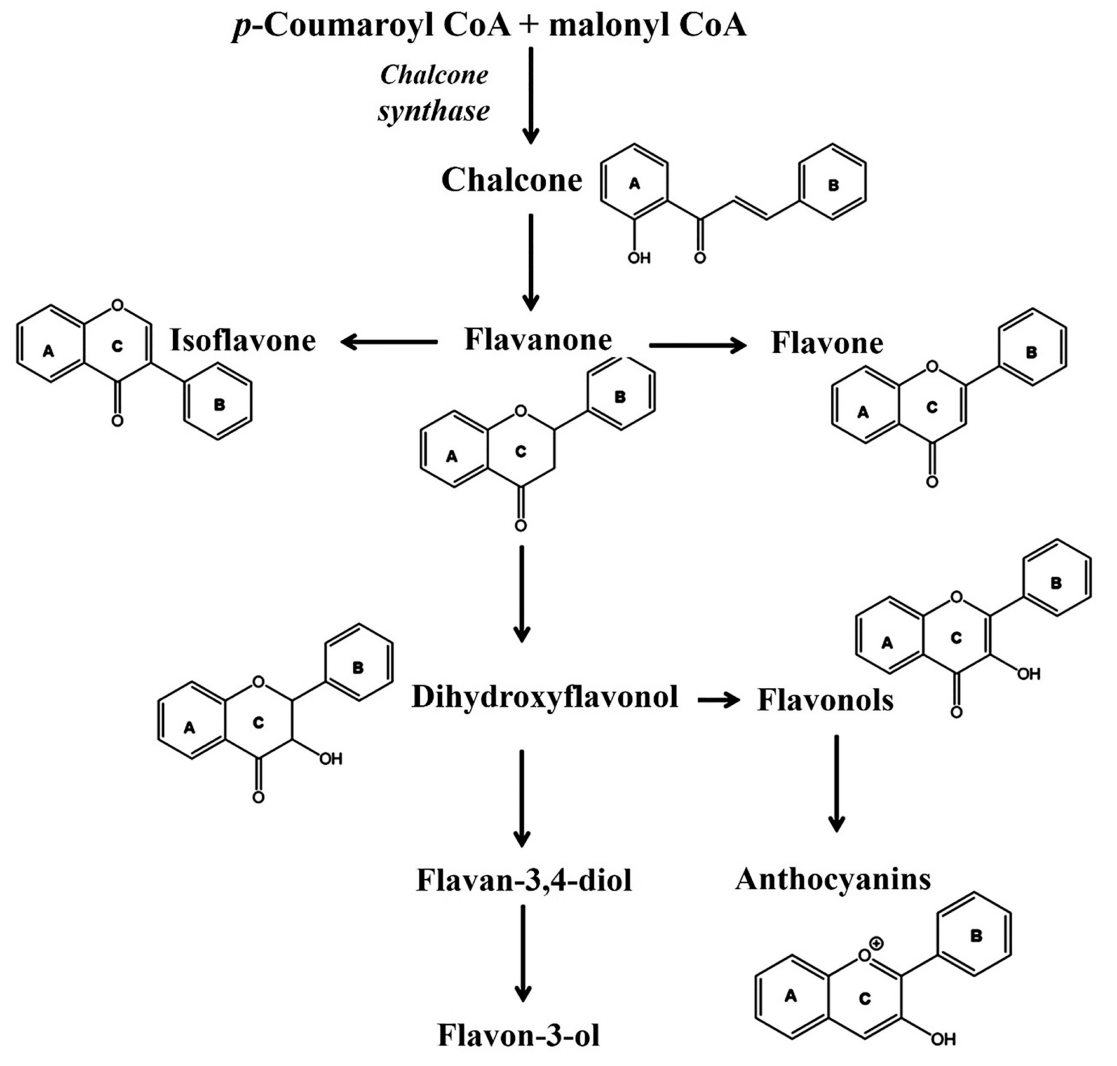
Schematic illustration representing biosynthesis and chemical structures of various flavonoids. Reproduced with permission from ref (63). Copyright 2021 MDPI.

The flavonoids, broadly categorized as anthocyanidins, flavanols, flavanones, flavones and flavonols (Figure 4), are mainly synthesized from 2′-hydroxychalcone produced through catalysis of p-coumaroyl CoA and malonyl CoA using chalcone synthase enzyme.^63^ Flavonoids have several biological properties that may assist in understanding why vegetables and fruits are associated with a lesser risk of lung cancer, along with antioxidant activity, inflammation inhibition, and antimutagenic and antiproliferative properties.^64^ Throughout the plant kingdom, the anthocyanidins (or anthocyanins) are omnipresent. Anthocyanins (ACNs) are typically glycosylated with glucose, galactose, arabinose, rutinose, and other plant sugars.^65^ Anthocyanidin (ACDs) refers to the aglycone forms of cyanidin, delphinidin, peonidin, petunidin, pelargonidin, and malvidin.^65^ Nonacylated monoglycosylated ACNs more competitively inhibited the cell growth of cancerous cells than triglycosylated ACNs and pelargonidin aglycones.^66^ On either hand, it has been recommended that a combination of several ACNs might be more efficient than individual ones in cancer therapy.^66^ A combined effect of suboptimum concentration levels of ACDs, vanquished the advancement of lung cancerous cells synergistically.^67^ In accordance with the available evidence, the absorption and elimination parameters were influenced mostly by the sugar moiety and the structure of the ACDs aglycone.^68^ Delphinidin, one of the many ACNs, has potent anticancer properties. Several research findings have shown that delphinidin treatment causes apoptotic cell death and seizes the cell cycle in different types of cancer.^69^ Two types of ACNs, cyanidin-3-glucoside and peonidin-3-glucoside(P3G), extracted from black rice significantly induced apoptosis and preferentially minimized proliferation and growth of HER2 positive breast cancer cells.^70^ Furthermore, P3G significantly reduced invasion of lung cancer cells and cyanidin-3-O-sambubioside extracted from fruits of the *Acanthopanax sessiliflorus* plant restrained angiogenesis and breast cancer cell progression.^67^

Flavonols, another subgroup of flavonoids, is used in remedying breast and gynecological cancer. Breast cancer is a heterogeneous disease because the underlying cause, response to treatment, and diagnosis of hormone receptor-positive and negative breast cancers differ.^7^ Women who consumed more flavan-3-ols had a reduced risk of ER2, but not ER+, breast cancer.^5^ One member of flavonol is epigallocatechin gallate (EGCG). EGCG suppressed the nicotine migration and growth of A549 lung cancerous cells, which was nicotine induced, as tobacco use is a familiar risk factor for lung cancer.^71^ The anticancer properties of EGCG may involve hormone activity modulation, due to which it is used in cancer treatment.^72^ Chemo preventive effects of EGCG involve preventing many signaling pathways in gastric and colon cancer.^73^ Furthermore, the cancer stem cell is involved in chemo resistance and cancer recurrence. EGCG has been shown to inhibit cancer stem cell advancement in breast, colorectal, neck, and head cancers in both *in vitro* and *in vivo* studies.^74−76^ Since androgen deficiency is a primary remedy for prostate cancer, it was confirmed that EGCG can biologically alienate the androgen level, indicating to downregulation of prostate tumor progression.^77^ Procyanidins also show a chemotherapeutic effect in colorectal cancer as an Caco-2 colon cancer cell.^78^

In the subgroup of flavanones, prenylated flavanones show potent anticancer activity in human prostate cancerous cell lines (PC-3 and DU-145) as well as a human hepatocarcinoma cell line.^79^ Both naringenin (NG)^80^ and hesperetin (HP)^81^ alleviated cancer cell proliferation, which lead to death of the cancer cells in gastric cancer cells. Besides this, NG inhibited cancer cell invasion in HCC cells by down-regulating multiple signaling pathways, which was also used in the treatment of HepG2 liver cancer cells as well as breast cancers.^82^ Because cancer cells have a high glucose uptake rate and utilization, this plays a crucial role in cancer development.^83^ As per one study, the antiproliferative effects of HP on breast cancer may be due to the reduction of glucose uptake.^84^ Furthermore, dietary HP demonstrated antiproliferative properties against chemical-induced colonic cancer and showed potent anticancer properties in cervical cancer cells.^67^ Another category consisting of apigenin (AG),^85^ chrysin,^86^ and luteolin (LT)^87^ exhibits potential effects in lung cancer cell apoptosis. In the group of flavonols, quercetin (QT) and kaempferol (KF), the two most ubiquitous flavonol aglycones, each have at least 279 and 347 unique glycosidic combinations.^23^ The most significant attribute of this flavonoid, QT, is its ability to showcase an effective antioxidant property and prevent cancer.^88^ QT’s ability to permeate through cellular membranes, due to its lipophilicity, causes inhibition of several intracellular pathways and are primarily involved in the prevention of various cancer types such as lung, liver, breast, prostate, colon, and cervical.^89^ It also exhibits anticancer activity through a variety of cellular signaling mechanisms and the ability to block enzymes that cause carcinogens to be produced.^90^ KF also shows high potential as a chemotherapeutic agent in cancer of the lung, gastric, colon and breast.^91^ Daidzein (DZ) induces apoptosis through mitochondrial apoptotic pathways in various cancer types, including breast, gastric, and hepatic, by varying the Bax/Bcl-2 ratio and triggering the caspase cascade.^92^ Genistein (GT) exhibits anticancer potential by inducing various mechanisms, including apoptotic induction, antimetastatic, cell cycle arrest, antiangiogenic, and anti-inflammatory effects.^93^

ACDs are commonly called anthocyanin, and out of the 31 anthocyanins, the most widely available are cyanidin, delphinidin, and pelargonidin, which along with their methylated derivatives account for 90% of the ACNs. Major dietary sources for this are berries, grapes, cherries, plums, and pomegranates.^94^ Catechins, also known as flavan-3-ols or flavanols, are a subtype of flavonoid. Flavanols are composed of simple monomers (catechins), which consist of epicatechin, epigallocatechin (EGC), EGCG, and polymers and/or oligomers, the latter two of which are identified as pro-ACDs or condensed tannins. Flavanols are mostly found in variety of foods, including apples, pears, legumes, tea, cocoa, and wine.^95^ NG from grapefruits and HP from oranges are the two most important flavanones.^96^ Prenylated flavanones, furanoflavanones, pyranoflavanones, and benzylated flavanones, for example, have distinct substitution patterns, resulting in many substituted derivatives in this subgroup.^96^ Flavones is another sub group of flavonoids and typically consist of AG and LT glycosides. Parsley and celery are excellent sources of flavones.^96^ Another important group of flavonoids is flavonols that consist of QT, KF, myricetin, isorhamnetin, galangin (GG). Major sources of flavonols are berries, apples, broccoli, beans, tea.^25^ GG is a flavonoid naturally present in oregano. Isoflavones have been classified as phytoestrogens due to structural similarities to estrogen. Representative groups of this subclass include GT and DZ from soy.^25^

### 3.2 Phenolic Acids

**Figure 5:**
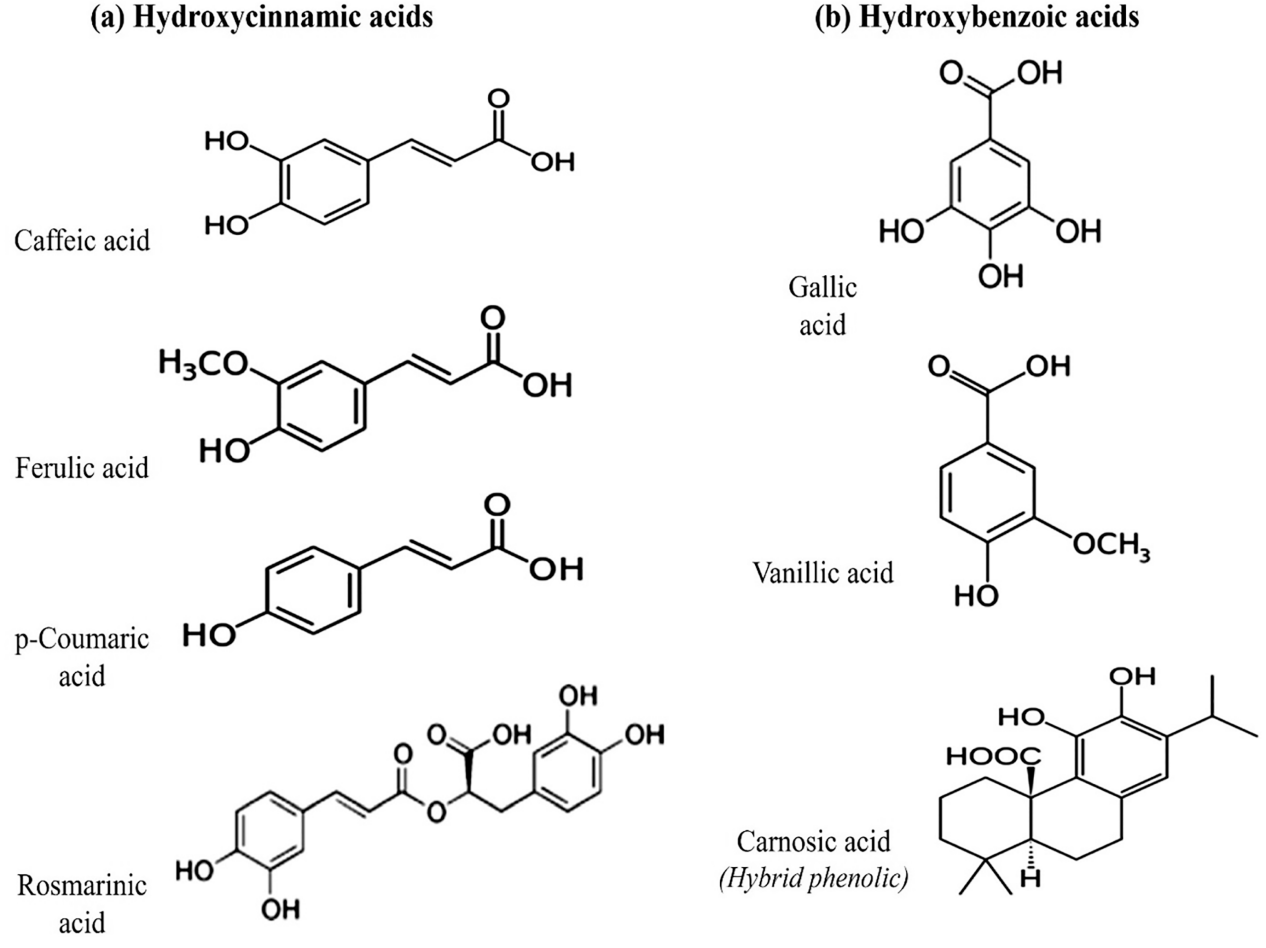
Chemical structures and configurations of various phenolic acids: (a) hydroxycinnamic acid derivatives and (b) hydroxybenzoic acid derivatives. Reproduced with permission from ref (97). Copyright 2021 MDPI.

Hydroxybenzoic acid (HBA) and hydroxycinnamic acid (HCA) are the two main groups classified under phenolic acids (PAs) (Figure 5). Hydroxybenzoic acid consists of ellagic acid (EA) and gallic acid (GA). These are found in grapes, walnuts, pomegranate, berries, wine, and green tea.^26,97^ HCA comprises of two major types chlorogenic acid and ferulic acid. The cereal grains, specifically the external surface of grain, are the main nutritional sources of ferulic acid.^26^

PAs are well-known for their beneficial effects as medicinal compounds in treating various diseases, including hyperglycemia, cardiovascular, neurodegenerative diseases, and cancer.^98^ EA acts as a potential chemotherapeutic agent in colon carcinogenic cells and suppresses breast cancer tumor development and angiogenesis as well as prostate malignant tumors invasion and motility.^99^ GA has a variety of therapeutic activities, including anticancer, antimicrobial, and anti-inflammatory.^100^ GAs manifest effective anticancer properties in various cancer cell lines like gastric, cervical, breast, and prostate cancers.^101^ Ferulic acid, a pro-oxidant at high levels, has gotten a lot of attention for its anticancer properties. As many cancer cells possess metal ions like copper, ferulic acid is selectively cytotoxic to them as compared to cancer cell lines.^102^

### 3.3 Stilbenes and Lignans

One more important class of polyphenols is natural stilbenes (STBs). Although the existence of natural STBs is very limited to only few plant varieties, numerous studies have been conducted on natural STBs due to the notable biological advantages of resveratrol (RVT), a key constituent of this class.^103^ Pterostilbene and piceatannol are few other representative members of this class. They are found in grapes, berries, and red wine.

**Figure 6:**
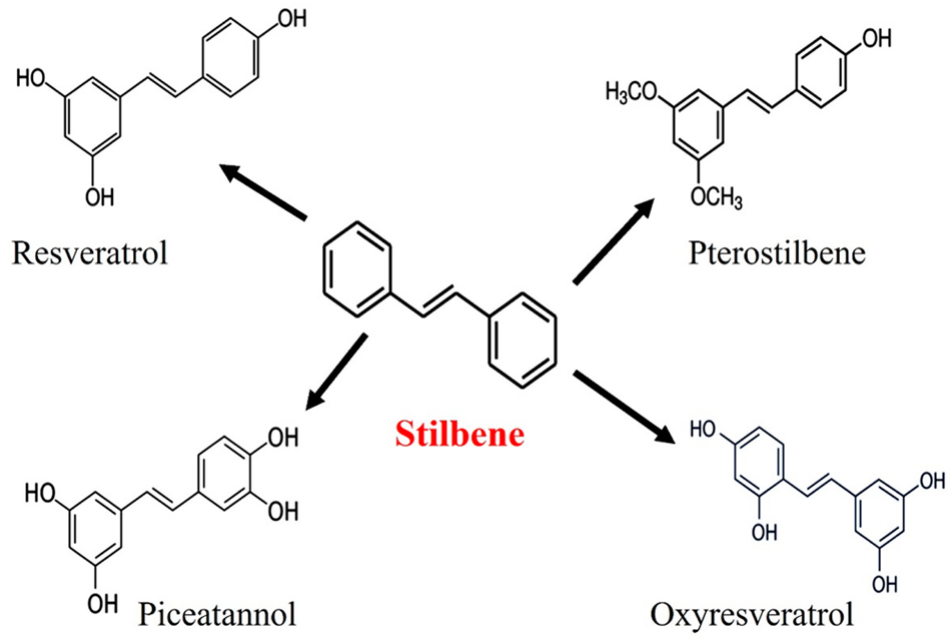
Chemical structures of various stilbenes. Reproduced with permission from ref (104). Copyright 2022 MDPI.

Natural STBs (Figure 6) are an essential member of nonflavonoid phytocompounds containing polyphenolic chemical configuration and indicated by the existence of a 1,2-diphenylethylene nucleus.^103,104^ One of the most studied STBs, RVT (3,4′,5-trihydroxy-*trans*-stilbene), exhibits potential anticancer properties preventing and treating different cancer types.^105^ Anticancer molecular mechanisms of RVT includes signaling pathways associated with cell propagation and genome uncertainty, receptor tyrosine kinases and extracellular growth factors, formation of multiprotein complexes and cellular metabolism, cytoplasmic tyrosine kinase signaling, signal transduction, apoptosis, immune surveillance, and hormone signaling.^106^

**Figure 7:**
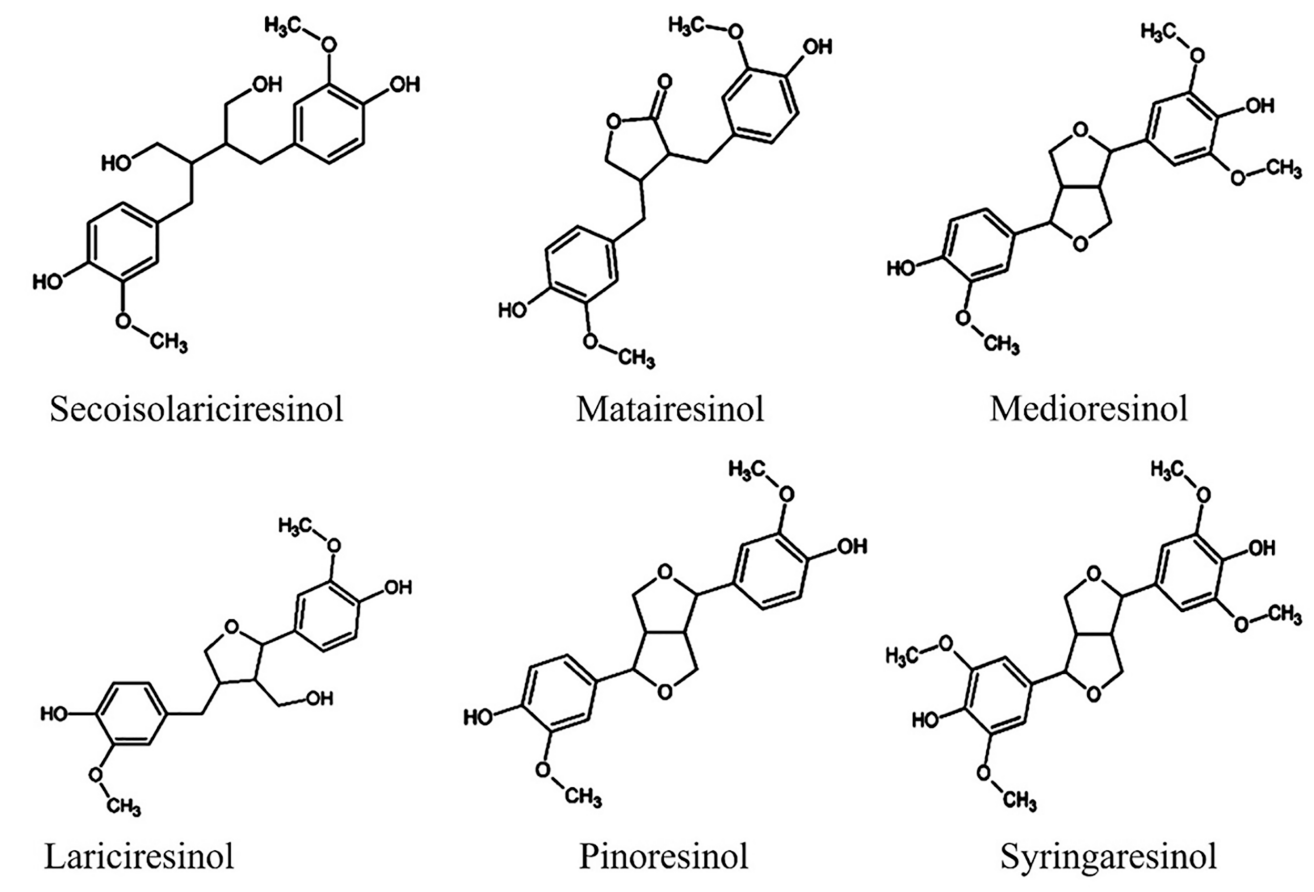
Chemical structures of various dietary lignans. Reproduced with permission from ref (107). Copyright 2018 MDPI.

Lignans (LIGs) due to their structural resemblance steroids are classified as phytoestrogens.^26^ Representative members of this group are secoisolariciresinol, matairesinol, medioresinol, lariciresinol, pinoresinol, syringaresinol (Figure 7).^107^ LIGs are found in many plants, including flaxseed and sesame.^26,108^ Studies have investigated the anticancer bioactivity of dietary lignans. LIGs have historically been linked to therapeutic benefits like cardioprotective effects, osteoporosis, and cancer management, especially hormone-related cancer like breast cancer.^109^

According to a study, RVT treatment increases the chemosensitivity of certain human nonsmall-cell lung carcinoma cells.^110^ RVT and RVT-conjugates show potent anticancer properties in gastric, colon, and breast cancer cells.^105^ Pterostilbene is primarily found in blueberries and is a naturally occurring dimethoxylated analogue of RVT.^111^ The lipophilicity, oral bioavailability, and biological half-life of pterostilbene are greater than those of RVT, showing better chemotherapeutic activity.^67^

## 4 Polyphenols-Loaded NanoeMulsion in Cancer Therapy

### 4.1 Flavonoids-Loaded Nanoemulsion in Cancer Therapy

Combining anticancer agents is a common practice to prevent toxic side effects, overcome cross resistance, and achieve a therapeutic effect that is synergistically enhanced.^112^ Flavonoids have intriguing anticancer effects, including the ability to reverse anticancer drug resistance and activity against cancer metastasis or growth-related mechanisms.^96^ Usually, incorporating a chemotherapeutic drug to flavonoids may increase therapeutic efficiency synergistically, leading to reduced dose and dosing frequency and so chances of toxicity is less or ngligble.^113^ In a study, researchers developed roselle extract-loaded NE (W/O) for pulmonary delivery. At pH 6.5 buffer, the roselle extract’s *in vitro* release was 44.7%; at pH 7.4, it was 40.7%. ACNs are used to treat lung cancer, but their use is limited due to physiological instability and lower oral bioavailability. This study created a stable roselle extract NE without them. The anthocyanin release rate was relatively slow, demonstrating its applicability as a nanocarrier for pulmonary delivery.^114^

**Figure 8:**
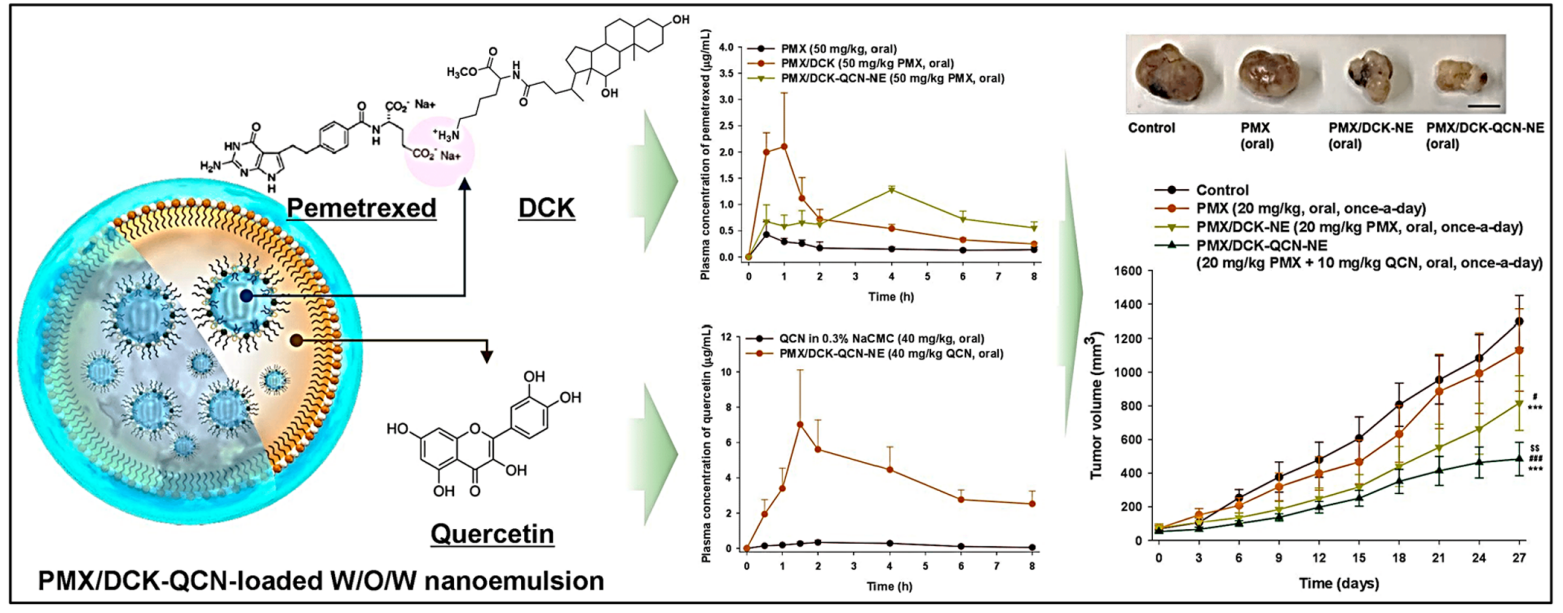
Schematic representation of the combined use of PTX/DCK and QRN in oral treatment had inhibitory effects on cell migration and proliferation in human lung carcinoma (A549). Reproduced with permission from ref (115). Copyright 2018 MDPI.

Pangeni et al.^115^ formulated W/O/W multiphase NEs for the simultaneous administration of Pemetrexed (PTX) and QT. This formulation had synergistic anticancer effects and improved oral absorption, as it increased the PTX’s permeation through the intestinal membrane and also enhanced the solubility of QT. The ideal NE had a droplet size, PDI, and zeta potential of 13.2 nm, 0.095 nm, and 3.99 mV, respectively. As shown in Figure 8, the combined use of PTX/N^α^-deoxycholyl-l-lysyl-methylester (DCK) and QT in oral treatment had inhibitory effects on cell migration and proliferation in human lung carcinoma (A549).^115^ Arbain et al.^116^ created a palm-based NE to deliver QT to the lung via an aerosol. Zetasizer results showed that the NE had a globular size of 106.1 ± 0.44 nm and a surface charge of −43.7 ± 3.57 mV.^116^ Samadi et al.^117^ conducted a novel approach to increase loading effectiveness and achieve QT sustained-release simultaneously. They loaded QT into an agarose-polyvinylpyrrolidone (PVP)-hydroxyapatite (HAp) hydrogel nanocomposite, increased loading efficiency by up to 61%, encapsulated it within W/O/W Nes, and exhibited potential cell apoptosis against the MCF-7 human breast cancer cell line.^117^

Hesperidin (HP)-loaded NEs (HP-NE) were prepared by Magura et al.,^118^ via spontaneous emulsification to improve the solubility, bioavailability, and efficacy of hesperidin for treating breast cancer using MCF-7 cell lines. Cell cycle seizure in the G2/M phase and death of cells through apoptosis were both brought on by treatment with HP-NE, indicating that HP-NE may be used as a therapeutic agent to treat breast cancer.^118^ The anticancer effect of optimized NG-loaded showed high stability, and controlled NG release from the NE was followed by an initial burst release. It also exhibited potential anticancer effect studies against A549 lung carcinoma cells. Thus, results indicated that the NE may be an appropriate route of drug administration to improve NG’s therapeutic potential of lung cancer.^119^

QT combined with vincristine exhibited a similar effect as that of verapamil, and docking studies revealed that QT binds specifically to ABCB1 in the analogous region. QT-loaded NE maintained its cytotoxic and cytostatic effects, and the unloaded-NE was capable to restrain efflux effects of ABCB1. The results indicated that QT might be a potential drug that can help in overcoming resistance in cancerous cells.^120^ In another study, researchers stated the chemotherapeutic potential of QT-loaded NE (∼50 nm) against human cancer cells in cytotoxicity activity (IC_50_ values; 24 h) order: HeLa > A549 > MIA PaCa-2.^29^ Altamimi et al.^121^ formulated LUT-loaded cationic NEs (LCNs) composed of bergamot oil (BO). The optimized formulation exhibited spherical appearance with particle size, PDI, and zeta potential values of 112 nm, 0.15, and +26 mV, respectively. The release rate of the drug was significantly improved after encapsulating to an emulsion system. Moreover, optimized formulation also exhibited an improved permeation flux, drug loading, and enhancement ratio than the drug suspension. Thus, this system can be used for targeting various cancer types, specifically breast cancer.^121^

**Figure 9:**
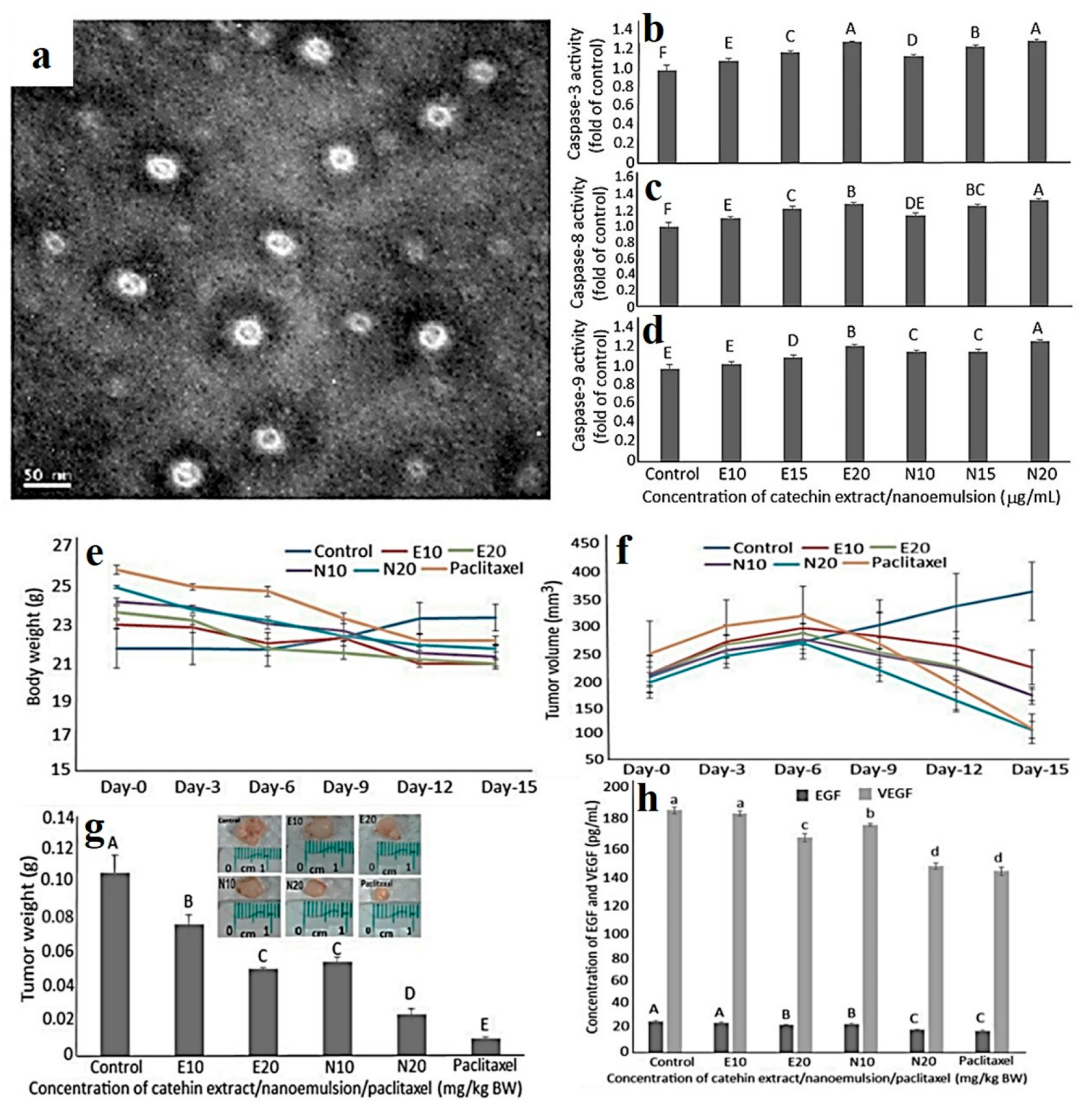
(a) Size and shape by transmission electron microscope. Effects of CE and CNE on (b) caspase-3 (b), caspase-8 (c), and caspase-9 (d) activities of prostate cancer cell DU-145 [CE treatment: E10 (10 μg/mL), E15 (15 μg/mL), and E20 (20 μg/mL); CNE treatments: N10 (10 μg/mL), N15 (15 μg/mL), and N20 (20 μg/mL). Data represented as mean ± s.d. (*n* = 3), with data bearing different capital letters (A–F) to denote significantly different values at *p* < 0.05. Effects of CE, CNE, and paclitaxel (PTX) on (e) body weight, (f) tumor volume, (g) tumor weight, and (h) serum EGF and VEGF levels of nude mice. [E10 (10 mg/kg BW) and E20 (20 mg/kg BW), with the same injection volume (0.2 mL), while N10 and N20 are CNE treatments at the same dose. For PTX treatment (0.2 mL and 10 mg/kg BW) was used. Data are shown as mean ± s.d. (*n* = 3), and capital letters (A–E) and small letters (a–d) denote significant different values at *p* < 0.05. Reproduced with permission from ref (122). Copyright 2021 MDPI.

Catechin NEs (CNE) fabricated from the Oolong tea leaves wastes were investigated against the prostate cancerous cell lines DU 145 and DU 145-induced tumors in animal model (mice) (Figure 9). CNE, compared to catechin extract (CE), exhibited greater stability with particle size, zeta potential, and entrapment efficiency 11.3 nm, −67.2 mV, and 83.4%, respectively. CNE effectively inhibited growth of DU 145 cells and upregulated caspase-8/-9/-3 levels, causing cellular apoptosis. CNE (20 g/mL) and PTX (10 g/mL) showed maximum therapeutic efficacy and inhibited tumor weight and volume.^122^ Tran et al.^123^ synthesized gold nanoparticles (GNs) using mountain ginseng (MG) and further loaded them to O/W NE (MG-GNNEs). MG-GNNEs significantly exhibited greater inhibitory effects against pro-inflammatory genes and proteins than blank MG-GNs and silydianin.^123^

Chitosan-coated LT-loaded NEs (CLNEs) were developed which exhibited improved permeation through the nasal mucosal membrane ex vivo with extended LT release up to 72 h *in vitro*. Pharmacokinetic studies results showed that the intranasal administration of CLNE showed a 10-fold upsurge in half-life and 4.4 times augmentation in drug’s biodistribution in brain tissues. These findings suggest that CLNE could act as a potential strategy for the management of brain disease/disorders like neuroblastoma.^124^*Arachis hypogaea* oil NE (ANE) was formulated and exhibited improved therapeutic efficacy against A549 lung carcinoma cells by inhibiting the cell viability and also antioxidant activity at 270.42 g/mL and 208.51 g/mL.^31^

### 4.2 Phenolic Acids-Loaded Nanoemulsion in Cancer Therapy

**Figure 10:**
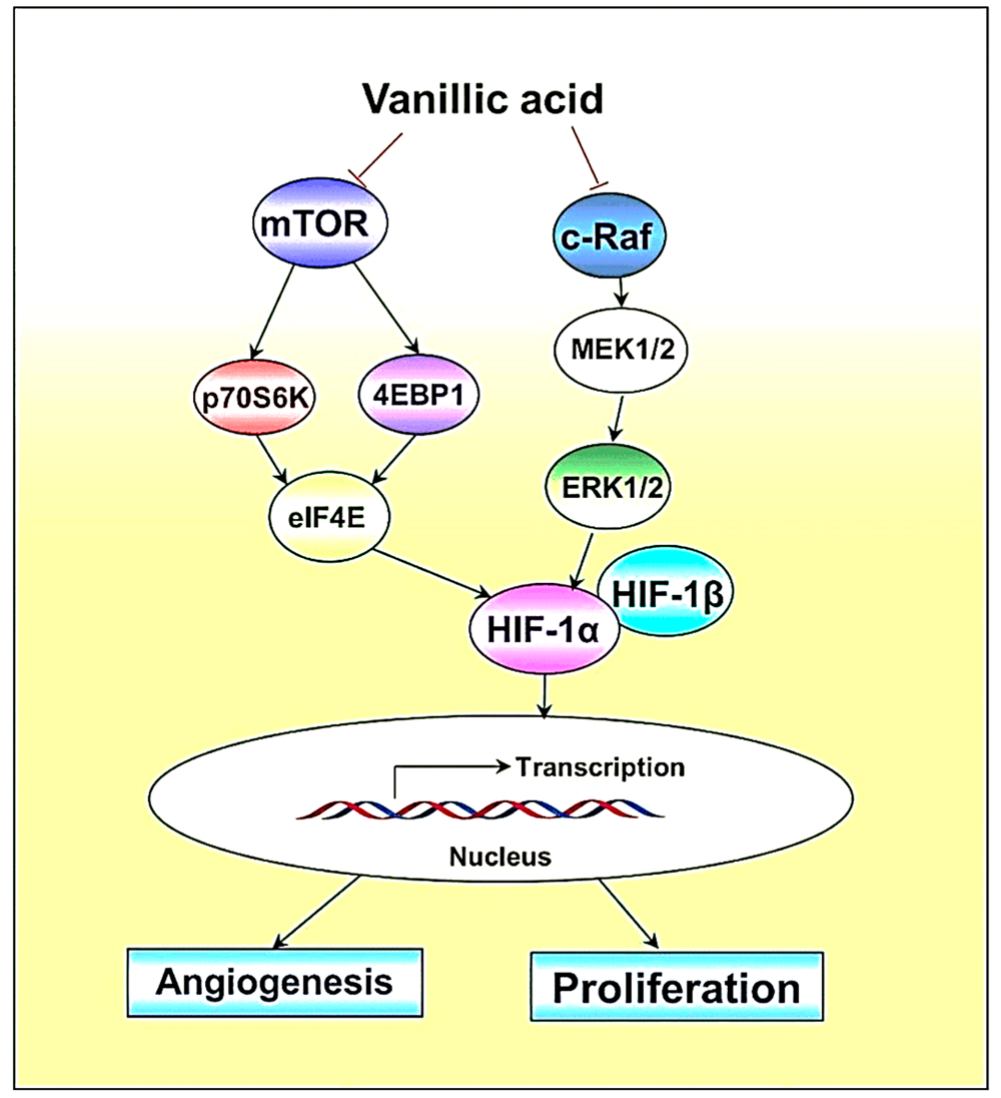
Schematic of proposed mechanism associated with vanillic acid: cell proliferation and angiogenesis inhibitory effect mainly by promoting downregulation of Hypoxia-Inducible Factor-1 alpha (HIF-1α) through inhibiting mTOR/p70S6*K*/4E-BP1 and Raf/MEK/ERK signaling pathways. Reproduced with permission from ref (125). Copyright 2019 MDPI.

PAs are plant secondary metabolites which in recent years have gained tremendous recognition as potential anticancer properties. These comprise of numerous phenolic compounds which contain one or more carboxylic acid groups.^98^ These can act as a promising anticancer agent by inducing apoptosis and reducing cell proliferation, by influencing diverse attributes of cancer including angiogenesis, tumor growth, cellular differentiation, and metastasis.^98^ Dietary vanillic acid (VA) inhibited Raf/extracellular signal-regulated kinase (ERK) kinase (MEK)/ERK, rapamycin-p70 ribosomal protein S6 kinase (p70S6K), and eukaryotic initiation factor 4E-binding protein-1 (4EBP1) pathways, when investigated *in vitro* on human colon cancer HCT116 cell lines (Figure 10). Moreover, they also distorted the tube formation and inhibited the expression of proteins VEGF and EPO, inhibiting angiogenesis. Cell cycle arrest at G1 phase and inhibition of proliferation was also observed in *in vitro* studies. Similar results were observed when VA was administered to a xenografted tumor model.^125^

Raviadaran et al.^126^ evaluated the therapeutic potential of tocotrienols (TT) and caffeic acid (CFA) loaded in W/O/W multiphase NEs, further coloaded with anticancer agent (cisplatin’ CP), against carcinoma cells. The prepared nanoformulation was successful in improving the apoptosis by 23.1% and 24.9% in the A549 and HepG2 cells, respectively. The production of ROS was also found to be doubled in HepG2 cells (30.2%) when compared to A549 cells (16.9%), while cell cycle arrest was observed at G0/G1 in both the cell lines used for the study.^126^

### 4.3 Stilbenes/Lignans-Based Nanoemulsion in Cancer Therapy

Stilbenes (STBs) compounds are found in numerous plant and tree species. They are commonly found in any medicinal plants. Compounds such as RVT, piceatannol, isorhapontigenin, pinosylvin, rhapontigenin, pterostilbene constitute stilbenes. They have shown various pharmacological activities including anticancer activity.^127^ Rinaldi et al. encapsulated RVT into an O/W NEs, which exhibited improved bioavailability and decreased cell viability in human T24 bladder cancer cells.^128^ Similarly, in another study, lipid-based self-nanoemulsifying delivery system encapsulating RVT was prepared, which exhibited improved cytotoxicity against the MCF-7 breast cancer cell line.^129^

**Figure 11:**
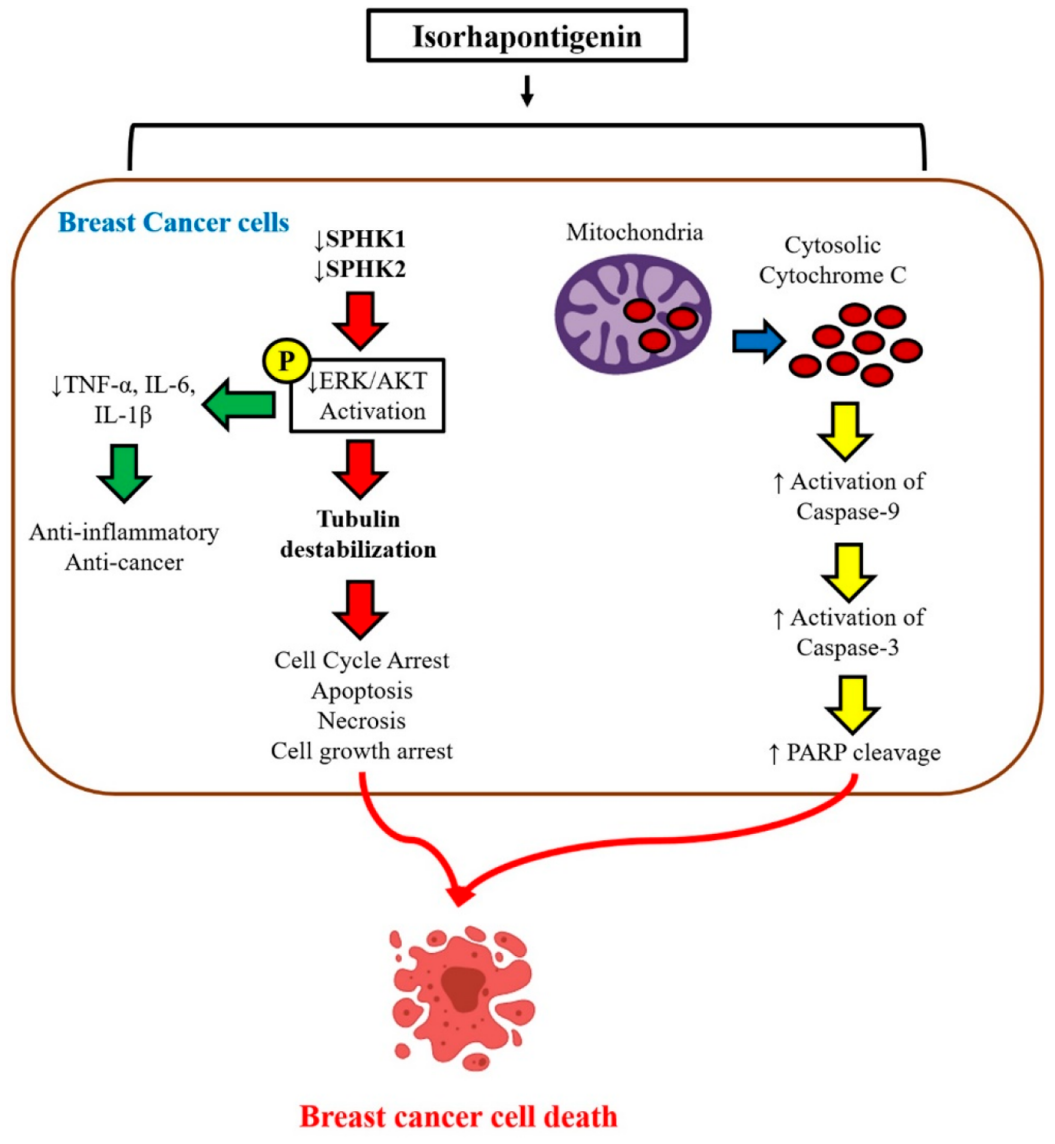
Graphical representation highlighting the anticancer effects of isorhapontigenin that was evaluated against the breast cancer (MCF7, T47D, and MDA-MB-231) cell lines. IRP-loaded nanoemulsions inhibited cancer cell growth and controlled tubulin polymerization using the MAPK/PI3K pathway. Reproduced with permission from ref (130). Copyright 2019 MDPI.

Isorhapontigenin (IRP), an analogue of RVT, also has shown promising results as a potential anticancer molecule. The anticancer effects of IRP (Figure 11) were evaluated against the MCF7, T47D, and MDA-MB-231 cell lines, where they inhibited cancer cell growth and controlled tubulin polymerization using the MAPK/PI3K pathway.^130^

Lignans, a class of polyphenols, are abundantly found in various plants, exclusively in seeds and whole grains. Antioxidant and anticancer properties of LIGs have been previously discussed. The antiproliferative property of secoisolariciresinol diglucoside, a LIG extracted from the plant *Linum usitatissimum* seeds, was evaluated on a MCF-7 cell line, where it inhibited tumor growth.^131^ Similarly, the anticancer properties of secoisolariciresinol and its metabolites secoisolariciresinol, enterolactone, and enterodiol have been discussed. In a study by Chen at al., incidence of mammary tumor was reduced in a rat model.^132^ Moreover, in rat models, morphological changes were observed in terminal bud end of mammary glands with administration of flax seed extracts. An enterolactone-induced apoptotic mechanism inhibited tumor growth in colo201 (human colonic carcinoma cell line), when investigated both *in vitro* and *in vivo*.

Demark-Wahnefried et al. investigated the use of flaxseed extracts for their antiproliferative effects on the antigens specific to prostrate and benign prostrate epithelium.^162^ Similarly, Inbaraj et al.^133^ synthesized the RVT-NEs, which exhibited a higher storage stability with a mean particle size, zeta-potential, and entrapment efficiency of 14.1 nm, −49.7 mV, and 95.5%, respectively. The NE inhibited the pancreatic cancer cells (BxPC-3) by down-regulating cyclin A, cyclin B, CDK1, and CDK2 expressions and up-regulating p53 and p21 expressions, leading to cellular apoptosis.^133^ Kotta et al.^134^ developed RVT-loaded thermosensitive hydrogels for effective delivery of RVT against breast cancertherapy, as it exhibited cytotoxic effects on breast cancer cells.^134^ Furthermore, few specific findings of anticancer effects and associated mechanism of polyphenols-loaded NEs against various cancer types is tabulated in Table 1, and the preclinical/clinical evidence of various polyphenols-loaded NEs have been enlisted in Table 2.

**Table 1:** Studies Showing the Anticancer Effects and Mechanism of Various Polyphenols on Specific Cancer Types

Polyphenol	Cancer (type)	*In vivo* or *in vitro* study	Major outcomes	Ref
Catechin	Prostate cancer	*In vitro*: the human prostate cancer cell line PC3	Inhibited tumor growth in PC3 cells	(135)
			Induced apoptosis by inhibiting Bcl-2 and activating caspases-3,8,9	
Puerarin	Breast cancer	*In vivo*: triple-negative breast cancer model	Puerarin NEs downregulated the production of ROS in the activated myofibroblast	(136)
Chalcones	Leukemia	*In vitro*: Monkey kidney epithelial cells (VERO) and acute lymphoblastic leukemia cells (L1210)	Chalcones-loaded NE induced higher toxicity and exhibited antileukemic effect in VERO cells	(137)
Catechin	Prostate cancer	*In vitro*: DU-145 cell line; *in vivo:* mouse model	Induced apoptosis by activating caspases-3,8,9, arrested (S- and G2/M)-cell cycle phases	(122)
Naringenin	Lung cancer	*In vitro*: A549 lung cancer cell line	Reduced the expression of Bcl2, increased activity of pro-apoptotic mediator’s caspase-3 and Bax	(119)
Genistein	Oral cancer	*In vitro*: human tongue squamous cell carcinoma (SCC-4 cell line) cells and pharyngeal squamous cell carcinoma (FaDu cell line) cells	GT-loaded NE improved the pharmacokinetic profile enhancing the drug’s bioavailability and prolonging release profile	(138)
Quercetin	Leukemia	–	Exhibited cytotoxic and cytostatic effects and bonded to ABCB1 at the similar region to that of verapamil	(120)
Hesperidin	Breast cancer	*In vitro*: MCF-7 cell line	Improved the drug’s solubility and enhanced bioavailability	(118)
			Arrested the cell cycle (G2/M-phase), further induced apoptosis by downregulating the expression of miR-22 and miR-155	
Silymarin	Hepatocellular carcinoma	*In vitro*: Human hepatocellular carcinoma HepG2 and Chang liver cell line	Silymarin-loaded NEs reduced the cell viability, while increasing ROS production and initiated chromatin condensation	(139)
			Pharmacokinetic parameters (a) decreased: viscosity and *T*_max_; (b) parameters increased: drug release, AUC, and *C*_max_	
Genkwanin	Colorectal cancer	*In vivo*: colitis-associated colorectal cancer (CAC) mouse models	Enhanced the drug’s solubility and intestinal permeability improving its bioavailability	(140)
			Induced apoptosis by decreasing the cytokines levels, inhibiting tumor growth	
Phenolic acids from date palm extracts	Breast and hepatocellular cancer	*In vitro*: MCF-7 and HepG2 cell lines	Reduced cell viability of treated MCF-7 and HepG2 cell lines	(141)
Phenols and Quercetin	Melanoma and lung adenocarcinoma	*In vitro*: human skin melanoma (G361) and lung adenocarcinoma (A549) cell line	Induced apoptosis and arresting cell cycle by exhibiting antiproliferative activity against G361 and A549 cell lines	(142)
Naringin	Lung adenocarcinoma	*In vitro*: A549 cell line	Exhibited improved cytotoxicity on the A549 cell line	(143)
			Bioaccumulation at secondary sites (potential sites for lung cancer metastasizing) were significantly higher	
Epigallocatechin gallate	Lung cancer	*In vitro*: H1299 cell line	Exhibited a improved antitumor activity, with MMP-2 and MMP-9 as the possible mechanisms for inhibition of tumor growth	(144)
Resveratrol	Breast cancer	*In vivo*: chick chorioallantoic membrane assay	Enhanced the anticancer and antiangiogenic activity	(129)
Resveratrol	Pancreatic cancer	*In vitro*: BxPC-3 cell line	Induced apoptosis in BxPC-3 cell line by upregulating the expressions of p53 and p21, while it downregulated the CDK1 and CDK2 expression	(133)
Pterostilbene	–	–	Improved the stability and solubility of pterostilbene	(145)
Enterolactone	Breast cancer	*In vitro*: MDA-MB-231 cell	Suppressed proliferation, relocation, and metastasis of MDA-MB-231 breast cancer cells	(146)

**Table 2:** Clinical Evidences of Few Polyphenol-Based Nanoemulsion Strategies in Cancer Therapy

Clinical Trial ID	Cancer types	Polyphenol	Comments	Phase/status	Ref
NCT03482401	Breast cancer	Polyphenol-rich dietary supplement containing 37 different phenolics and 2 methylxanthines	Inhibited breast tissue-occurring metabolites proliferation in p53-wild-type MCF-7 cells by inducing cell cycle arrest, senescence, and apoptosis via p53/p21 activation	NA/completed	(147)
NCT01912820	Prostate cancer	QT	Green tea’s anticancer properties were discovered to be enhanced by QT	Phase I/completed	(148)
NCT00949923	Breast cancer	Epicatechins, EGC, EGCG	Polyphenols resulted in reduction in proliferation and increase in apoptosis post-treatment	NA/completed	(149)
NCT05758571	Interstitial pneumonia in cancer	EGCG	Exhibited pro-apoptosis, antifibrosis, anti-inflammatory, antitumor, and metabolic effects through modulating a variety of intracellular signaling cascades	Phase I/II	(150)
NCT03751592	Squamous nonsmall cell lung cancer	Chlorogenic acid	–	Phase I/II	(151)
NCT05306002	Breast and ovarian cancer syndrome	–	Following 1 to 3 months of oral supplementation, chromosome breakage was reduced	NA	(152)
NCT02029352	Melanoma	EGCG	Exerted cytotoxic effect on skin cancer cells, induced apoptosis, and suppressed cell development	Phase II/III	(153)
			EGCG inhibited catenin signaling, which is a key component of the WNT pathway		
NCT01360320	Colorectal cancer	EGCG	–	Phase II	(154)
NCT01496521	Esophageal squamous cell carcinoma	Tea polyphenols	–	Phase III	(155)
NCT02728349	Glioblastoma	Chlorogenic acid	–	Phase I	(156)

## 5 Conclusion and Future Perspective

Cancer is one of the major causes of death worldwide, even the second foremost cause related to noncommunicable diseases. Even though, a significant reduction of 31% in cancer-related deaths is observed in the previous 30 years, possibly due to improved lifestyle choices, the disease still poses a significant threat to public health systems across the globe. Natural polyphenols are organic compounds obtained from plants that are identified by the presence of two or more phenol units in their structure.^157^ A plethora of research has been conducted on polyphenols to investigate potential health advantages, including antioxidant, anticancer, antimicrobial, diabetes, antiviral, cardioprotective effects, neurodegenerative disease, and aging.^158^ The polyphenols can block cell cycle events, induce apoptosis, and modify signaling pathways to eliminate cancer cells. Moreover, polyphenols regulate the actions of enzymes that promote the malignant cells growth. Recent investigations have come up with findings showing association of the natural polyphenols with anticancer effects mostly due to inhibiting DNA interaction, antiangiogenic, and antimetastatic effects against various cancer types.^159,160^

NEs possess some desirable characteristics such as (1) they are usually transparent giving them a pleasing appearance, (2) naturally resistant to the typical destabilizing mechanisms that are present in emulsions, (3) offer numerous chances to increase the oral bioavailability of very lipophilic drugs.^33,35^ Since NEs allow for real-time cancer surveillance with minimal invasion and destruction, their usage as imaging agents is rapidly growing. Conventional imaging techniques, such as magnetic resonance imaging, ultrasound, and X-ray tomography, all depend on labeling a target NE with a fluorophore or radioactive isotope.^33^ Vaccine carriers in NE formulations that target tumors are also in the more recent stages of research. As was previously mentioned, antigens and other macromolecules can be delivered via NEs and trigger an advantageous immune system response that is particular to the antigen. Thus, to develop a highly precise interaction, NEs allow a extended circulation duration and uptake of cells having the specific antibody for that antigen over their exterior surficial phase, or vice versa.^161^ In the future, additional epidemiological findings using polyphenol-mediated biomarkers are desired and can be helpful to measure the anticancer effect of dietary polyphenols on cancer types. More extensively, randomized clinical trials must be conducted to offer more dependable evidence. Furthermore, the bioavailability of polyphenols should be assessed and improved with focused consideration for their safety.
